# Autochthonous *Leishmania siamensis* in Horse, Florida, USA

**DOI:** 10.3201/eid1809.120184

**Published:** 2012-09

**Authors:** Sarah M. Reuss, Mark D. Dunbar, Maron B. Calderwood Mays, Jennifer L. Owen, Martha F. Mallicote, Linda L. Archer, James F.X. Wellehan

**Affiliations:** University of Florida, Gainesville, Florida, USA (S.M. Reuss, M.D. Dunbar, J.L. Owen, M.F. Mallicote, L.L. Archer, J.F.X. Wellehan, Jr.);; and Florida Vet Path, Inc, Bushnell, Florida, USA (M.B. Calderwood Mays)

**Keywords:** Leishmania, leishmaniasis, cutaneous, equine diseases, parasites, zoonoses, United States

**To the Editor:** *Leishmania siamensis*, a recently described species, was identified as the cause of autochthonous visceral leishmaniasis in 2 men in southern Thailand (*1*,*2*). Cutaneous leishmaniasis has been reported in horses in Europe and South America. Lesions in horses are solitary or multiple nodules that are often ulcerated and most commonly occur on the head, pinnae, legs, and neck. Other clinical signs are usually absent. In South America, biochemical characterization has identified *L. braziliensis* in horses (*3*). Leishmaniasis has been reported in horses in Puerto Rico (*4*), and equine leishmaniasis has been described, but no reports have been published, in the United States. *L. infantum* has been reported in equine cutaneous leishmaniasis in Europe (*5*). A report from central Europe recently identified an organism with 98% nucleotide identity over the ITS (internal transcribed spacer) 1 region to *L. siamensis* as the cause of cutaneous leishmaniasis in 4 horses (*6*). *L*. *siamensis* was also identified in a case of cutaneous bovine leishmaniasis in Switzerland (*7*).

In August 2011, a 10-year-old, 505-kg Morgan horse mare in Florida, USA, with no history of travel outside the eastern United States was evaluated at the University of Florida for an ulcerated mass in the left pinna. When, 6 months earlier, the owner had noticed the mass, it was ≈1 cm in diameter, firm, raised, and covered with hair. Three months later, the ear was unchanged, and the mare was successfully impregnated. Over the subsequent 2 months, the mass began to grow and ulcerate. At that time, veterinary consultation was obtained and a biopsy performed. Histologic study showed that the dermis was hyperplastic and diffusely infiltrated with neutrophils, macrophages, and lymphocytes (Technical Appendix Figure 1). Numerous intracytoplasmic protozoal organisms with a small nucleoid and smaller kinetoplast most consistent with *Leishmania* sp. were observed in macrophages. No treatment was pursued. After 45 days, the mare was seen at the University of Florida because of progression of the lesions. The mass on the internal aspect of the pinna was 6 cm × 3 cm and ulcerated, and 3 new firm 1 cm–diameter nodules were observed on the outer pinna of the same ear. Multiple soft, less-defined, 1 cm to 3 cm–diameter nodules were observed along both sides of the neck, shoulders, and withers. No other abnormalities were observed on physical examination or thoracic and abdominal ultrasound, and lymph nodes were not enlarged. Ultrasound confirmed a ≈90 day viable pregnancy. Complete blood count and plasma chemistry were within normal limits.

Tissue aspirates were taken of the multiple ear lesions and of the nodules along the neck and shoulder. From the ulcerated lesion, marked mixed, predominantly neutrophilic inflammation was seen, and rare neutrophils and macrophages contained intracellular protozoal organisms consistent with *Leishmania* sp. amastigotes (Technical Appendix Figure 2). These organisms were 4–5 µm in diameter and round with pale basophilic cytoplasm. They had an eccentrically placed, basophilic, oval nucleus and a small, basophilic, rod-shaped kinetoplast oriented perpendicular to the long axis of the oval nucleus. No organisms were seen in any other aspirates.

Fresh tissue was submitted for PCR, which has been determined suitable for detecting Old World leishmaniasis in dogs (*8*). Results were negative. Given the clear clinical, cytologic, and histologic evidence for cutaneous leishmaniasis, additional consensus PCR was performed as described (*9*), targeting the ITS1 region. Direct sequencing was performed by using the BigDye Terminator Kit (Applied Biosystems, Foster City, CA, USA) and analyzed on ABI 3130 automated DNA sequencers (Applied Biosystems) at the University of Florida Interdisciplinary Center for Biotechnology Research Sequencing Facilities (Gainesville, FL, USA). The resultant sequence was 310 bp after primers were edited out. Sequence alignment yielded a genotype with 99% identity to the first *L. siamensis* isolate (GenBank accession no. EF200012) and 100% identity to 2 more recently submitted sequences from human visceral leishmaniasis isolates from Thailand (GenBank accession nos. JQ001751 and JQ001752) (Technical Appendix). The sequence was submitted to GenBank (accession no. JQ617283).

The mare delivered a stillborn foal at 350 days’ gestation. Histopathology did not reveal any infectious organisms in the fetal tissues; however, the chorioallantois showed moderate villous atrophy, which was presumed to be the cause of fetal death. One month after foaling, the mare’s cutaneous lesions were 90% resolved.

Because the mare in this report was born in the United States and had never left the country, this case appears to be autochthonous. Mode of transmission is unknown. Phlebotomine sand flies found in Florida include *Lutzomyia shannoni*, *Lu. cubensis*, *Lu. vexator*, and *Lu. cruciata*. *Lutzomyia* sp. are competent vectors of *Leishmania* spp. in other areas of the world. However, the vector for reported cases of *L. siamensis* in other regions has not been identified. Although leishmaniasis is infrequently diagnosed in any species in Florida, models have shown that with climate change, the range of sand flies and accompanying leishmaniasis in North America is expected to expand substantially (*10*). This report raises many avenues for further investigation: the prevalence of leishmaniasis in horses in the United States, understanding of the life cycle and vectors, and the zoonotic potential of this *Leishmania* species.

Technical Appendix Multiple alignment with Fast Fourier Transform of *Leishmania siamensis* ITS1 sequences; histologic section and fine needle aspirate of ulcerated mass from a horse with cutaneous leishmaniasis.

## References

[1] Sukmee T, Siripattanapipong S, Mungthin M, Worapong J, Rangsin R, Samung Y, A suspected new species of *Leishmania*, the causative agent of visceral leishmaniasis in a Thai patient. Int J Parasitol. 2008;38:617–22. 10.1016/j.ijpara.2007.12.003 18262531

[2] Suankratay C, Suwanpimolkul G, Wilde H, Siriyasatien P. Autochthonous visceral leishmaniasis in a human immunodeficiency virus (HIV)–infected patient: the first in Thailand and review of the literature. Am J Trop Med Hyg. 2010;82:4–8. 10.4269/ajtmh.2010.09-0434 20064986 PMC2803500

[3] Barbosa-Santos EGO, Marzochi MCA, Urtado W, Queirós F, Chicarino J, Pacheco RS. Leishmaniasis disseminated by *Leishmania braziliensis* in a mare (*Equus caballus*): immunotherapy and chemotherapy assays. Mem Inst Oswaldo Cruz. 1994;89:217–20. 10.1590/S0074-02761994000200018 7885248

[4] Ramos-Vara JA, Ortiz-Santiago B, Segalès J, Dunstan RW. Cutaneous leishmaniasis in two horses. Vet Pathol. 1996;33:731–4. 10.1177/030098589603300619 8952039

[5] Solano-Gallego L, Fernández-Bellon H, Serra R, Gállego M, Ramis A, Fondevila D, Cutaneous leishmaniosis in three horses in Spain. Equine Vet J. 2003;35:320–3. 10.2746/042516403776148336 12755438

[6] Müller N, Welle M, Lobsiger L, Stoffel MH, Boghenbor KK, Hilbe M, Occurrence of *Leishmania* sp. in cutaneous lesions of horses in Central Europe. Vet Parasitol. 2009;166:346–51. 10.1016/j.vetpar.2009.09.001 19800739

[7] Lobsiger L, Müller N, Schweizer T, Frey CF, Wiederkehr D, Zumkehr B, An autochthonous case of cutaneous bovine leishmaniasis in Switzerland. Vet Parasitol. 2010;169:408–14. 10.1016/j.vetpar.2010.01.022 20153118

[8] Gaskin AA, Schantz P, Jackson J, Birkenheuer A, Tomlinson L, Gramiccia M, Visceral leishmaniasis in a New York foxhound kennel. J Vet Intern Med. 2002;16:34–44. 10.1111/j.1939-1676.2002.tb01604.x 11822802

[9] el Tai NO, Osman OF, el Fari M, Presber W, Schönian G. Genetic heterogeneity of ribosomal internal transcribed spacer in clinical samples of *Leishmania donovani* spotted on filter paper as revealed by single-strand conformation polymorphisms and sequencing. Trans R Soc Trop Med Hyg. 2000;94:575–9. 10.1016/S0035-9203(00)90093-2 11132393

[10] González C, Wang O, Strutz SE, Gonazalez-Salazar C, Sanchez-Cordero V, Sarkar S. Climate change and risk of leishmaniasis in North America: predictions from ecological niche models of vector and reservoir species. PLoS Negl Trop Dis. 2010;4:e585. 10.1371/journal.pntd.0000585 20098495 PMC2799657

