# Supplementary material for: Autochthonous Leishmania siamensis in Horse, Florida, USA
Source: Emerg Infect Dis. 2012 Sep;18(9):1545–7. doi: 10.3201/eid1809.120184 (PMC3437729; doi:10.3201/eid1809.120184)
Supplement: Technical Appendix — Multiple alignment with Fast Fourier Transform of Leishmania siamensis ITS1 sequences; histologic section and fine needle aspirate of ulcerated mass from a horse with cutaneous leishmaniasis. [file 12-0184-Techapp-s1.pdf]

Article DOI: http://dx.doi.org/10.3201/eid1809.120184

Autochthonous *Leishmania siamensis* in Horse, Florida, USA

Technical Appendix

Multiple alignment with Fast Fourier Transform of Leishmania siamensis ITS1 sequences. Nucleotides with differences are shown in red.

```
Horse FL          ATTACA-CCAAAAAACATACAGG-TAGAGA-GTAGTAGAATACATCTACTCGGGGAGGCATGTTTTTTCCG-ATATGCCTTTCCCACATACACAAACACAGCAATATATATGT
Bovine|GQ281282   ATTACA-CCAAAAAACATACAGGCTAGAGA-GTAGTAGAATACATCTACTCGGGGAGGCATGTTTTTTCCG-ATATGCCTTTCCCACATACACAAACACAGCAATATATATGT
Horse|GQ281281    ATTACA-CCAAAAAACATACAGGCTAGAGA-GTAGTAGAATACATCTACTCGGGGAGGCATGTTTTTTCCG-ATATGCCTTTCCCACATACACAAACACAGCAATATATATGT
Human|JQ001751    ATTACA-CCAAAAAACATACAGG-TAGAGA-GTAGTAGAATACATCTACTCGGGGAGGCATGTTTTTTCCG-ATATGCCTTTCCCACATACACAAACACAGCAATATATATGT
Human|JQ001752    ATTACA-CCAAAAAACATACAGG-TAGAGA-GTAGTAGAATACATCTACTCGGGGAGGCATGTTTTTTCCG-ATATGCCTTTCCCACATACACAAACACAGCAATATATATGT
Human|GQ293226    ATTACA-CCAAAAAACATACAGG-TAGAGA-GTAGTAGAATACATCTACTCGGGGAGGCATGTTTTTTCCG-ATATGCCTTTCCCACATACACAAACACAGCAATATATATGT
Human|GQ226034    ATTACA-CCAAAAAACATACAGG-TAGAGA-GTAGTAGAATACATCTACTCGGGGAGGCATGTTTTTTCCG-ATATGCCTTTCCCACATACACAAACACAGCAATATATATGT
Human ref|EF200012 ATTACACCCAAAAAACATACAGG-TAGAGAGGTAGTAGAATACATCTACTCGGGGAGGCATGTTTTTTCCGTATATGCCTTTCCCACATACACAAACACAGCAATATATATGT

Horse FL          ATATATATACGTATATTGCTATACCCAAAAACCATACCGTAAAAAGCAAAAAGGCCGGTCGACGCCAAATGCCGCGCGTATACAGTGGAAAAGTCCGTTCGTTACGGCTCTTT
Bovine|GQ281282   ATATATATACGTATATTGCTATACCCAAAAACCATACCGTAAAAAGCAAAAAGGCCGGTCGACGCCAAATGCCGCGCGTATACAGTGGAAAAGTCCGTTCGTTACGGCTCTTT
Horse|GQ281281    ATATATATACGTATATTGCTATACCCAAAAACCATACCGTAAAAAGCAAAAAGGCCGGTCGACGCCAAATGCCGCGCGTATACAGTGGAAAAGTCCGTTCGTTACGGCTCTTT
Human|JQ001751    ATATATATACGTATATTGCTATACCCAAAAACCATACCGTAAAAAGCAAAAAGGCCGGTCGACGCCAAATGCCGCGCGTATACAGTGGAAAAGTCCGTTCGTTACGGCTCTTT
Human|JQ001752    ATATATATACGTATATTGCTATACCCAAAAACCATACCGTAAAAAGCAAAAAGGCCGGTCGACGCCAAATGCCGCGCGTATACAGTGGAAAAGTCCGTTCGTTACGGCTCTTT
Human|GQ293226    ATATATATACGTATATTGCTATACCCAAAAACCATACCGTAAAAAGCAAAAAGGCCGGTCGACGCCAAATGCCGCGCGTATACAGTGGAAAAGTCCGTTCGTTACGGCTCTTT
Human|GQ226034    ATATATATACGTATATTGCTATACCCAAAAACCATACCGTAAAAAGCAAAAAGGCCGGTCGACGCCAAATGCCGCGCGTATACAGTGGAAAAGTCCGTTCGTTACGGCTCTTT
Human ref|EF200012 ATATATATACGTATATTGCTATACCCAAAAACCATACCGTAAAAAGCAAAAAGGCCGGTCGACGCCAAATGCCGCGCGTATACAGTGGAAAAGTCCGTTCGTTACGGCTCTTT

Horse FL          CTCTCTCGCGGGTGTGTGTGTGGATAACGGCTCACATAACGTGTCGCGATGGA-TGACTTGGCTTCCTATTTCGTTGAAGAACGCAGTA
Bovine|GQ281282   CTCTCTCGCGGGTGTGTGTGTGGATAACGGCTCACATAACGTGTCGCGATGGA-TGACTTGGCTTCCTATTTCGTTGAAGAACGCAGTA
Horse|GQ281281    CTCTCTCGCGGGTGTGTGTGTGGATAACGGCTCACATAACGTGTCGCGATGGA-TGACTTGGCTTCCTATTTCGTTGAAGAACGCAGTA
Human|JQ001751    CTCTCTCGCGGGTGTGTGTGTGGATAACGGCTCACATAACGTGTCGCGATGGA-TGACTTGG---------------------------
Human|JQ001752    CTCTCTCGCGGGTGTGTGTGTGGATAACGGCTCACATAACGTGTCGCGATGGA-TGACTTGG---------------------------
Human|GQ293226    CTCTCTCGCGGGTGTGTGTGTGGATAACGGCTCACATAACGTGTCGCGATGAA-TGACTTGG---------------------------
Human|GQ226034    CTCTCTCGCGGGTGTGTGTGTGGATAACGGCTCACATAACGTGTCGCGATGGACTGACTTGG---------------------------
Human ref|EF200012 CTCTCTCGCGGGTGTGTGTGTGGATAACGGCTCACATAACGTGTCGCGATGGA-TGACTTGGCTTCCTATTTCGTTGAAGAACGCAGTA
```

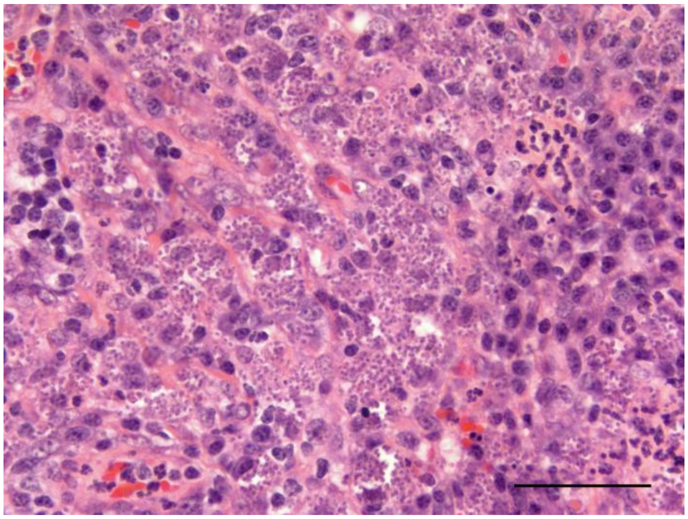

Figure 1. Histologic section of an ulcerated mass from a horse with cutaneous leishmaniasis. Macrophage cytoplasms contain myriad protozoa, each with a small nucleoid and a smaller kinetoplast. Original magnification ×50.

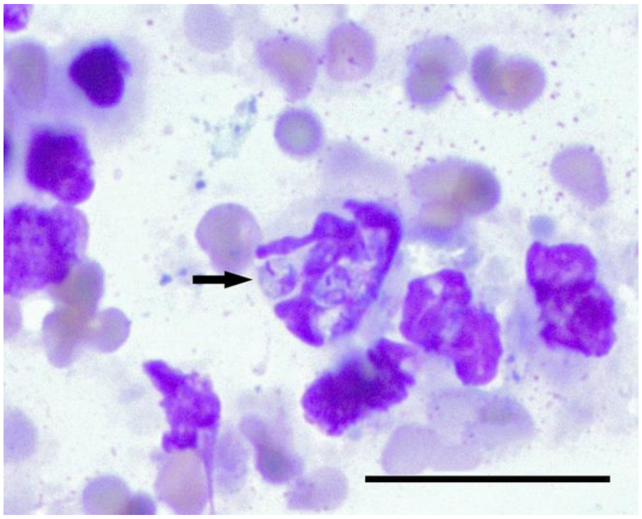

Figure 2. Fine needle aspirate of an ulcerated mass from a horse with cutaneous leishmaniasis exhibiting an intracellular amastigote (arrow). Original magnification ×100.
